# Computer-assisted methods for molecular structure elucidation: realizing a spectroscopist's dream

**DOI:** 10.1186/1758-2946-1-3

**Published:** 2009-03-17

**Authors:** Mikhail Elyashberg, Kirill Blinov, Sergey Molodtsov, Yegor Smurnyy, Antony J Williams, Tatiana Churanova

**Affiliations:** 1 Advanced Chemistry Development, Moscow Department, 6 Akademik Bakulev Street, Moscow 117513, Russian Federation; 2 Novosibirsk Institute of Organic Chemistry, Siberian Division, Russian Academy of Sciences, 9 Akademik Lavrent'ev Av., Novosibirsk, 630090 Russian Federation; 3 ChemZoo Inc., 904 Tamaras Circle, Wake Forest, NC, 27587, USA

## Abstract

**Background:**

This article coincides with the 40 year anniversary of the first published works devoted to the creation of algorithms for computer-aided structure elucidation (CASE). The general principles on which CASE methods are based will be reviewed and the present state of the art in this field will be described using, as an example, the expert system Structure Elucidator.

**Results:**

The developers of CASE systems have been forced to overcome many obstacles hindering the development of a software application capable of drastically reducing the time and effort required to determine the structures of newly isolated organic compounds. Large complex molecules of up to 100 or more skeletal atoms with topological peculiarity can be quickly identified using the expert system Structure Elucidator based on spectral data. Logical analysis of 2D NMR data frequently allows for the detection of the presence of COSY and HMBC correlations of "nonstandard" length. Fuzzy structure generation provides a possibility to obtain the correct solution even in those cases when an unknown number of nonstandard correlations of unknown length are present in the spectra. The relative stereochemistry of big rigid molecules containing many stereocenters can be determined using the StrucEluc system and NOESY/ROESY 2D NMR data for this purpose.

**Conclusion:**

The StrucEluc system continues to be developed in order to expand the general applicability, provide improved workflows, usability of the system and increased reliability of the results. It is expected that expert systems similar to that described in this paper will receive increasing acceptance in the next decade and will ultimately be integrated directly to analytical instruments for the purpose of organic analysis. Work in this direction is in progress. In spite of the fact that many difficulties have already been overcome to deliver on the spectroscopist's dream of "fully automated structure elucidation" there is still work to do. Nevertheless, as the efficiency of expert systems is enhanced the solution of increasingly complex structural problems will be achievable.

## Background

The potential of creating computer-assisted methods for the structure elucidation of *new *organic compounds was first discussed in the second half of the past century. Structure elucidation commonly combines information extracted from several forms of spectra. The molecular formula of the substance is generally derived from a mass-spectrum and structural hypotheses are deduced from spectral data which may usually include NMR, IR, UV, *etc*. spectra. The distinctive feature of this approach is the inference of the structure of an unknown compound that is absent from spectral libraries, i.e. without employing reference structures and their associated spectra. Later, qualitative spectral analysis without reference data was extended to quantitative spectral analysis in optical spectroscopy [1]. The solution of such problems can be facilitated by the retrieval of reference data in combination with logical-combinatorial processing of the data. A new area of investigation was developed that is now referred to as Computer-Aided Structure Elucidation (CASE). CASE was applied initially to "small molecules" as distinct from biological macromolecules and biopolymers.

The first reports devoted to CASE systems were published by four independent groups of researchers exactly forty years ago [2-5]. Since the publication of these seminal reports an extensive literature regarding computer methods of structure elucidation has been produced. From the inception of CASE methods, attention has been directed to the creation of artificial intelligence or "expert" systems (ES) based on the analysis of 1D ^1^H and^13^C NMR data in combination with MS and IR spectra. The first studies of CASE development were described in a series of reviews [6,7] and monographs [8-10]. In spite of the efforts of many scientific groups no system capable of elucidating large complex molecules was delivered during the first 20 years of intensive efforts. The primary reason for failure was the lack of structural information that could be retrieved from 1D NMR spectra to use as input to the structure generator, the kernel of any expert system. The first two decades of CASE development should, nevertheless, be considered as very fruitful since a general strategy was established and the core algorithms for structure elucidation and verification necessary to deliver expert systems were defined. The results obtained during this period have been reviewed [11-13], and the general methodology of expert systems as tools for molecular structure elucidation have been reviewed by Elyashberg, *et al*. [14]. An article entitled "Fully automated structure elucidation – a spectroscopist's dream comes true" [15]) suggested an effective method of application of extensive database (ca. 500 000 items) containing large fragments supplied with their ^13^C NMR spectra. Unfortunately, this approach also failed to realize the spectroscopist's dream which seems hardly to be achieved in a visible future.

A new area of analytical chemistry – "chemometrics" was born during the same period and CASE was considered as an integral part of chemometrics in this time. Depending on the field of application and the mathematical approaches used two branches were distinguished in chemometrics: quantitative and qualitative. *Quantitative chemometrics *is based on continuous mathematics and the major areas in this field include multivariate calibration, pattern recognition, mathematical mixture resolution, etc. The primary focus of these quantitative approaches is to quantitative chemical analysis and spectrum processing. *Qualitative chemometrics *is based on discrete mathematics mainly on mathematical logic, graph theory and combinatorial analysis. These methods form the basis of the methodologies developed for Computer-Aided Structure Elucidation.

A new epoch in CASE development was initiated in the nineties when the first reports dedicated to expert systems based on 2D NMR spectra were published [16-21]. Nowadays 2D NMR data can be routinely generated, even in automation, and a multitude of data are available as inputs to CASE systems – e.g. HSQC (HMQC), ^1^H-^1^H COSY (TOCSY) and HMBC methods. The present capabilities of 2D NMR expert systems to perform structure elucidation and verification was recently reviewed by Elyashberg *et al *[22]. It is worth noting that previously CASE systems were associated with chemometrics but leading chemometricians now assign the term "chemometrics" to quantitative chemometrics only and the journal of *Chemometrics and Laboratory Intelligent Systems *does not accept articles devoted to CASE systems.

This article intends to show how CASE systems are approaching the goal of providing fully automated structure elucidation using CASE systems. The main principles on which these systems are based will be discussed. Our own work regarding the development of the expert system Structure Elucidator [23-30] will be used to demonstrate all stages of structure elucidation by modern computer-aided methods. Advantages of CASE methods are demonstrated on some examples of structure revision.

## Results and Discussion

### General principles of the CASE systems

#### A molecule as a "machine" for coding structural information

**Figure 1:**
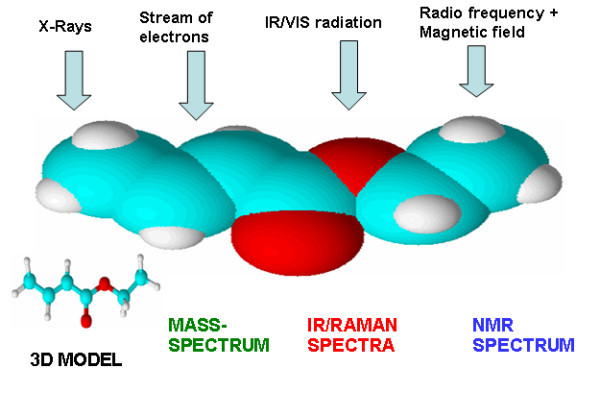
**The molecule as a cipher machine for coding structural information**.

CASE expert systems are based on the same general cognitive principles common to the properties of particles belonging to the atomic and sub-atomic world. In order to extract information regarding some property of a particle it is necessary to stimulate the particle using electromagnetic radiation or a stream of particles and to analyze the response signal. In those cases where we want to extract information about the structure of a molecule we excite the system with electromagnetic radiation over a wide frequency range using electrical and magnetic fields and streams of electrons or ions. As a result we obtain UV-VIS, IR, Raman, NMR and mass-spectra or even, when X-rays are used, the 3D structure model of a molecule (see Fig. 1.). In this case the molecule under analysis acts like a specific coding machine (cipher machine) which codes structural information into each kind of spectrum using its own code. The goal of a researcher is to crack these codes and extract the maximum structural information achievable. Figuratively speaking the CASE problem can be formulated in the following way: create a decoding machine capable of decoding the structural information contained in one or more spectra. It should be noted that structural information is coded into different types of spectra at different levels of complexity. For instance, depending on experimental conditions mass-spectrometry can produce spectra containing a lot of structural information, but extraction of the details can be very complicated. Nevertheless, MS usually can deliver the molecular mass and, with improved resolution and accuracy in recent years it is now fairly simple to determine the molecular formula for the molecule under study, a key parameter necessary for molecular structure elucidation.

An IR spectrum can provide valuable information about the presence or absence of certain functional groups but communicates very little about their environment in the molecule. The richest structural information can be extracted from NMR spectra since the environment of a given magnetically active nucleus (^1^H, ^13^C, ^15^N, etc.) can be revealed through the chemical shift value and the spin-spin couplings with neighboring nuclei. NMR spectra are therefore considered as a primary source of structural information.

**Figure 2:**
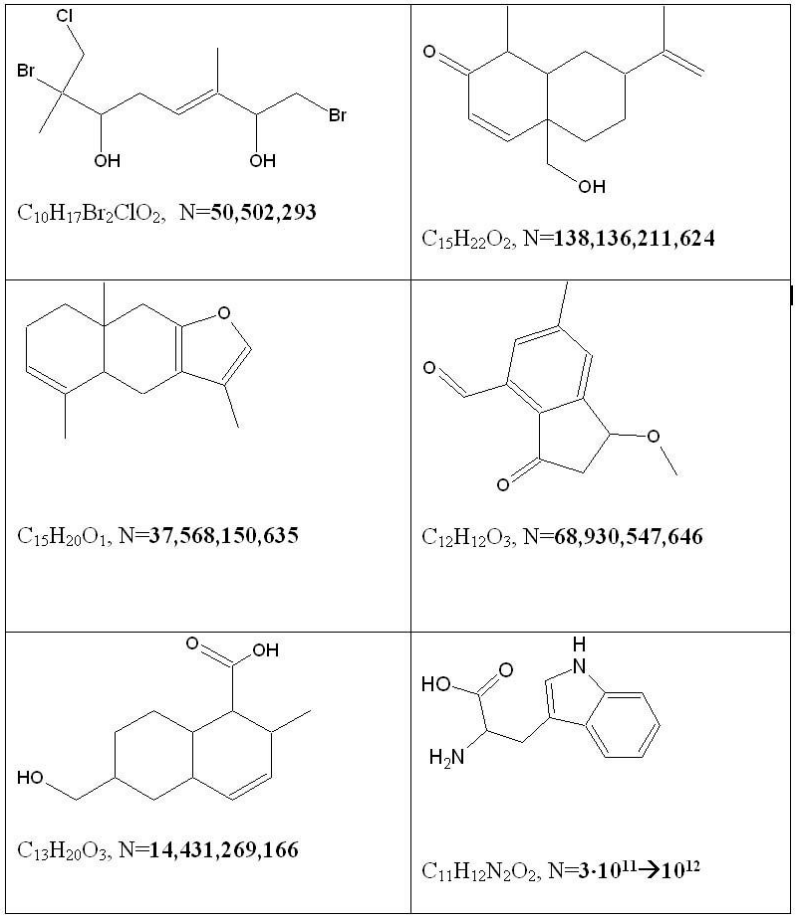
**Structures of some small molecules and theoretical numbers of isomers (N) corresponding to their molecular formulae**.

The development of computer-based methods of structure elucidation requires that isomerism be taken into account and, indeed, is the primary challenge. Fig. 2. displays the structures of a series of small molecules of natural products and the number of potential structural isomers calculated by us in our work [29]. The figure shows that even the simplest structures can have hundreds of millions if not billions of isomers. The number of isomers associated with the structures of medium-sized complex organic molecules can be estimated as about 10^20^–10^30 ^isomers (on the order of Avogadro's number). Although the number of isomers is huge those corresponding to a given molecular formula do make up a *countable *and *finite *set. We can conclude that the general CASE strategy utilizes processes to eliminate "superfluous" isomers from the full isomer set by imposing different structural constraints produced from the molecular spectra and *a priori *information (sample origin, chemical rules, etc.). A successful result depends on the screening and rejection of N-1 structural formulae that do not comply with the experimental data and systematic constraints applied.

It is possible to suggest the following straightforward approach to solving the CASE problem: 1) generate all isomers from a given molecular formula; 2) impose different structural constraints one by one until the initial number of isomers reduces down to one. This approach can actually be used but only in those cases where the unknown molecule is small (not more than 15 skeletal atoms). For larger molecules the number of isomers and the associated processing time are so huge that the problem cannot be solved, a situation referred to as a "combinatorial explosion"). To reduce the dimension of the problem it is necessary to introduce molecular fragments which account for a significant number of the skeletal atoms. The main attention of researchers engaged in developing CASE systems was focused on the creation of the optimal methods for selecting appropriate fragments and elaboration algorithms for structure generation from fragments and free atoms.

During the first period of expert system development (when 1D NMR, IR and MS, etc. spectra were used), it was realized that fragment selection can be automated on the basis of a "characteristic features" concept. This concept can be formalized by establishing the main axioms and hypotheses used for interpretation of molecular spectra [6,8]. The two following statements are typical:

1. If a molecule contains fragment ***A*_*i*_**, then the characteristic spectral features of the fragment *X*_1_, *X*_2_,...*X*_*j*_,...*X*_*m *_will be observed in the spectrum.

2. If a feature *X*_*j *_*is *observed in the spectrum, then the molecule contains at least one of the fragments **A**_*i*_(*X*_*j*_), **A**_*k*_(*X*_*j*_), ... **A**_*l*_(*X*_*j*_).

The first statement is an "*axiom*" because it reflects the experimentally established fact. The second one is a hypothesis because not all conceivable fragments are known and the *X*_*j *_feature can be implicated by any unknown fragment. These statements can be formalized using mathematical logic and all possible combinations of fragments that may be present in a molecule under analysis can be found as a solution to the corresponding logical equation [3,6,8]. The creation of a set of axioms and hypotheses necessary for solution of a given problem is equivalent to the creation of some particular *axiomatic theory*. To obtain a valid solution to the problem (i.e. a manageable output structural file containing the correct structure) the set of axioms must be *true*, *complete *(in definite sense) and *consistent*. A clear understanding of the described nature of the problem is crucial for correct interpretation of the solution obtained for a particular problem.

The aim of the expert system is obviously to extract the maximum amount of structural information from the available spectral data. In principle, the structural information can be easily quantified [14]. Assume that all *N *conceivable isomers corresponding to the molecular formula are stable enough, and let *p*_*i*_, be the probability that the *i*th (1 ≤ *i *≤ *N*) isomer is the genuine structure. Before solving the problem, all isomers are equiprobable and *p*_*i *_= 1/*N*. Then, the entropy *E*_0_characterizing the initial uncertainty of the solution can be determined by Shannon's formula [31]*E*_0 _= *log*_2_*N*. The entropy of the valid solution *E*_*v *_containing *n *structures will be *E*_*v *_= *log*_2_*n*. Obviously, *E*_*v *_<*E*_0_. Then the quantity of information *I*_*v *_obtained as a result of the solution of the problem can be expressed as the difference between the initial and final entropies:

*I*_*v *_= *E*_0_-*E*_*v *_= *log*_2_*N *- *log*_2_*n *= *log*_2_*(N/n)*.

If *n *= 1 then the structure of the unknown compound is unambiguously determined and the total quantity of structural information *I*_0 _obtained as a result of the solution of the problem can be expressed as *I*_0 _= *E*_0 _= *log*_2_*N*. Hence, the extraction of new structural information is accompanied by a decrease in the number of isomers that meet constraints imposed by the experimental data (spectra), and the process of structure elucidation reduces to the successive imposition of structural constraints on the number of possible isomers up to the extraction of complete structural information. If complete structural information is taken as 100% then the fraction of complete structural information *q *extracted from spectra as a result of the solution of the problem can be estimated by the ratio

*q *= (*I*_*v*_/*I*_*o*_) × 100 = (1 - log_2_*n*/log_2_*N*) × 100.

Thus, for each particular problem, the most informative method is that allowing for the extraction of the largest fraction of total structural information and the elimination of the maximum number of incorrect structures.

#### Structural constraints

In general, structural constraints can be arbitrarily set in two forms: affirmative (positive) and negative. Among the affirmative constraints are the specification of the hybridization of carbon atoms, the obligatory neighborhoods of some carbon atoms with heteroatoms, the enumeration of fragments that can (or must) be present in the molecule and the specification of the permissible sizes of cycles, etc. Negative constraints form a system of prohibitions: the prohibition of neighborhoods with certain heteroatoms, the prohibition of the presence of particular fragments, sizes of cycles, specific bond orders, etc. The requirement of the best match between the calculated spectrum of the expected structure and the experimental spectrum can be considered as the most rigid constraint. Calculated spectra impose constraints not only on characteristic spectral features, but on all spectral features without exception.

NMR data are certainly the most informative spectra for the purpose of molecular structure elucidation while ^13^C NMR spectra are more informative still when compared with ^1^H NMR spectra. However, their combined use yields a synergistic effect and is especially pronounced in 2D NMR spectra. 2D NMR spectra provide information useful not only for the structure elucidation of the molecular skeleton but also for the determination of the relative stereochemistry of the molecule and calculation of its 3-D model.

"Negative" structural constraints, which are introduced because of the absence of some characteristic features in the spectrum, are commonly more informative than positive constraints. For example, the absence of signals in the region 150–200 ppm in the ^13^C NMR spectrum suggests with a high probability that the carbonyl group is absent in the molecule, whereas the presence of a signal in this region can also be accounted for by the presence of other groups (C = N, C = S, C = C-O, etc.).

A calculation of the quantity of structural information obtained from each constraint and ranking constraints by their contributions into solving a particular problem is, in principle, feasible. These quantitative estimations are only possible for small molecules however.

#### Main stages of the computer-aided structure elucidation

**Figure 3:**
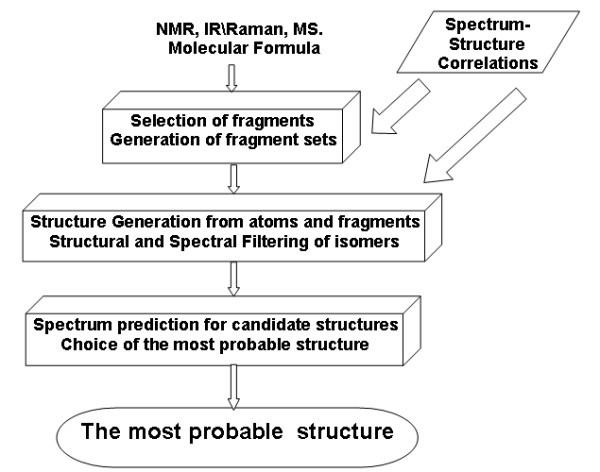
**Flow diagram of a CASE system**.

During the 1970s researchers established a general workflow for the CASE process (Fig. 3.). Based on the reasoning outlined above the sequence of operations that forms the basis of expert system operation is both clear and natural.

Any available combination of molecular spectra may be used as initial data. First, different positive structural constraints, mainly fragments, are determined from the spectra and input into the program. Using this information and the molecular formula, the system generates isomers. Obviously, it is necessary to obtain only the isomers that meet all of the imposed constraints and spectral features observed in the experimental spectra of the unknown compound. The fulfillment of structural constraints is ensured by the generation algorithm, which exhaustively yields all structures that meet the constraints. The compliance of the structures with the observed spectral features is verified using spectro-structural correlations in spectra of different nature. They form a set of spectral filters. The filters involve the most typical molecular fragments (functional groups) together with variation intervals of spectral features typical for these fragments. Constraints that are a system of prohibitions resulting from the rules of organic chemistry, stereochemistry, and a priori knowledge about the studied compound are added into the structural filter. The fragments included in filters are searched for in each structure. Structures containing fragments that are not confirmed by the spectra are excluded from the output structural file. The final step of structure filtration is the check of the proximity of the calculated and experimental spectra for each structure. The structure for which the predicted spectra are closest to the experimental spectra is selected as the most probable.

It should be noted that this flow diagram (Fig. 3.) has essentially remained valid until recently in spite of the fact that the algorithms used to realize different stages of the structure elucidation process were continuously varied and improved during the last forty years.

**Figure 4:**
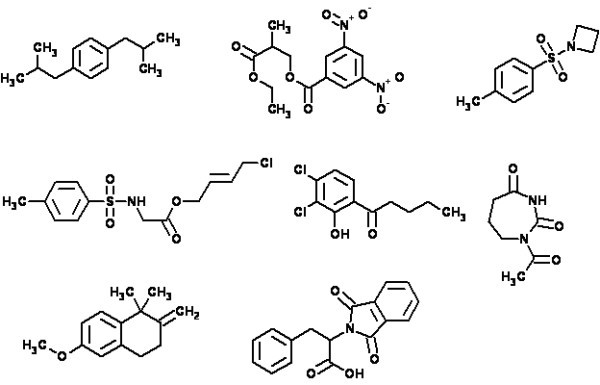
**Examples of structures identified with the aid of the X-PERT program **[32].

As mentioned above, when 1D NMR spectra were used the execution of procedures described by the flow diagram were successful only for modest sized molecules. Typical examples of molecules recognized by the 1D expert system X-PERT [32] are shown in Fig. 4.

#### Usage of structural information carried by 2D NMR spectra

The molecule size limitations were overcome when 2D NMR data became available to second generation expert systems. We will briefly consider the properties of structural information obtained from 2D NMR spectra. 2D NMR spectra of various forms can be acquired in various ways. In general they exhibit spin-spin couplings for each magnetic nucleus interaction with other magnetic nuclei in a molecule. This allows us, in principle, to elucidate the molecular structure as though the elucidation were performed *ab initio *without the use of spectrum-structure correlations. Commonly, the following types of 2D experiments are used [33]:

^1^H-^13^C heteronuclear correlation (for example, HSQC (Heteronuclear Single-Quantum Coherence));

^1^H-^1^H homonuclear correlation (for example, COSY (Homonuclear Correlation SpectroscopY));

^1^H-^13^C long-range heteronuclear correlation (for example, HMBC (Heteronuclear Multiple-Bond Correlation)).

HSQC spectra exhibit spin-spin couplings between the ^13^C_*i *_nucleus from the C_*i*_H_n _(group (*n *= 1–3) and the protons bonded to this atom, which makes it possible to establish the relationship between the chemical shift in the ^13^C spectrum of the C_*i *_atom and the chemical shifts in the ^1^H spectrum from protons bonded to this atom. COSY spectra commonly exhibit peaks caused by the interaction between protons separated by two or three bonds from each other (though longer-range couplings are possible). These signals correspond to spin-spin coupling constants ^2^J_HH _(a geminal interaction in a CH_2 _group) and ^3^J_HH _(a vicinal interaction in the C-l(H-l)- C-2(H-2) fragment).

Useful structural information is obviously provided by correlation peaks detected from vicinal interactions between hydrogen atoms. If the vicinal interaction of H-l and H-2 protons is revealed in the COSY spectrum, it is said that a *connectivity *with a length of one C-C bond occurs between the corresponding C-l and C-2 atoms. The richest information is involved in HMBC spectra. These spectra exhibit spin-spin couplings between protons and ^13^C nuclei separated by *two or three *bonds from each other. The difficulty of interpreting HMBC spectra is that conventional HMBC experiments do not distinguish ^2^J_CH _and ^3^J_CH _coupling peaks. Therefore, a length of one to two C-C bonds is assigned to HMBC connectivities. In general the larger the number of COSY and HMBC connectivities the more structural information (constraints) is extracted from the spectra.

As well as the 2D NMR experiments mentioned above, there is a series of other 2D NMR techniques (TOCSY, NOESY, ROESY, etc.), which elucidate long-range proton-proton interactions. For example, TOCSY interactions are transmitted through a chain of neighboring protons. Interactions through-space can be detected using NOESY and ROESY spectra and they allow conclusions to be drawn regarding the stereochemistry of the molecule.

In expert systems employing 2D NMR spectra it is assumed by default that COSY connectivities have a length equal to one C-C bond and the length of HMBC connectivity is specified within one or two C-C bonds. These parameters correspond to the distances between interacting spins most commonly observed in practice. These assumptions make up a set of "axioms" in 2D NMR spectroscopy.

COSY and HMBC spectra can, for certain physical reasons [33,34], also exhibit signals corresponding to the coupling of spins separated by distances larger than the default options of the program. These correlations are called *nonstandard correlations *[28]. If, for example, the COSY spectrum exhibits a significant cross peak from the interaction of H-l and H-3 protons from the C-l(H-l)-C-2(H-2)-C-3(H-3) fragment, the program by default takes it as an indication that the C-l and C-3 atoms are adjacent. Taking into account that a particular chemical shift is assigned to each carbon atom, a contradiction appears with the other data. As a result, the problem either has no solution (number of structures *n *= 0) or the solution is invalid. In our example, the contradiction is eliminated by the extension of the (C-l)-(C-3) connectivity by one C-C bond.

However, in practice the problem is to elucidate whether 2D NMR data involve nonstandard connectivities and, if their presence is found, which of the "suspicious" connectivities must be extended and by how many bonds. Note that the total number of 2D NMR correlations is commonly from several tens to hundreds. Unfortunately, no reliable experimental methods currently exist that allow the unambiguous identification of nonstandard correlations. Our studies demonstrated [30] that in some cases the number of nonstandard correlations in 2D data approaches 20 and the deviation from the "standard" connectivity length can be from one to three bonds. Our experience has shown that the probability of the presence of at least one nonstandard connectivity in a 2D NMR spectrum for a molecule under study was about 50% [29].

Since 2D data involve correlations of variable length even under those conditions when the default options of the program adequately represent the specific features of spin couplings in the analyzed molecule, the problem of structural interpretation of the spectra reduces to deriving the structure from *fuzzy *data. The situation is dramatically complicated if at least one nonstandard correlation occurs among the initial data: the data become *contradictory*. A large uncertainty is also introduced by signal overlap in the ^13^C and ^1^H spectra. As a result of the presence of nonstandard correlations, their quantity, and the true length of each of them are *a priori *unknown, we arrive at a conclusion that, in the general case, initial information can be not only *fuzzy*, but also *contradictory *and *indefinite*. It should also be noted that some expected peaks may not appear in the 2D spectra. If a deficiency of hydrogen atoms occurs in the molecule, the problem sometimes becomes unsolvable without the introduction of particular fragments. Then, the initial 2D information is also *incomplete*.

Taking into account the properties of initial 2D NMR data, it is unreasonable to expect that in the *general case *an expert system can elucidate the molecular structure from 2D NMR spectra in a completely *automated *mode. However many problems, as our experience demonstrates [25-27], can be solved in an unattended mode. Since fully-automated solutions are uncommon it was necessary to develop interactive methods for computer-assisted molecular structure elucidation taking into account the properties of 2D NMR information. We developed these methods based on the systematization of experience accumulated as a result of successful and unsuccessful attempts to solve a large number of complex problems. These methods and algorithms were implemented in the expert system Structure Elucidator (StrucEluc) [23-30], which, evidently, is currently the most advanced system available. Using the example of this system we will consider the methodology of molecular structure elucidation from 2D NMR spectra with the use of computer-assisted methods.

### Expert System Structure Elucidator

#### Common Mode of System Operation

##### Knowledgebase of the system

The system was developed for the purpose of elucidating the structure and the relative stereochemistry of a complex organic molecule from a combination of spectral data but specifically using 2D NMR spectra. The system knowledge can be divided into two categories. The *factual *knowledge contained within the system consists of the following components:

• A Complete Structure Library (CSL) containing about 400,000 molecules accompanied by their ^13^C and ^1^H NMR spectra,

• A Fragment Library (FL) containing more than 1.5 million fragments together with their ^13^C NMR subspectra,

• A library containing 175,000 structures and their assigned ^13^C and ^1^H NMR spectra intended for the prediction of ^13^C and ^1^H chemical shifts from the structural formula of molecules.

The axiomatic knowledge includes:

• An Atom Property Correlation Table (APCT) which is used for setting atom hybridization and details regarding possible neighboring heteroatoms. The library contains atom-centered fragments with variation intervals of chemical shifts of the central carbon atom in the ^13^C NMR spectra and the intervals of chemical shifts of the protons attached to the central atom.

• Correlation tables for spectral-based structure filtering. The library contains the most widespread functional groups with intervals of their characteristic features in the NMR and IR spectra.

The reliability of this axiomatic knowledge i.e. the correspondence between the spectral ranges and the structural fragments included in the correlation tables was thoroughly checked. The filtering of both correlation tables through the CSL subset containing 280,000 structures has shown that 98% of structures passed through the verification procedures [29].

The molecular formula or molecular mass of the analyzed compound, HSQC, HMBC, and COSY spectra, as well as 1D ^13^C and ^1^H NMR spectra are used as initial data. If the ^13^C NMR spectrum cannot be recorded because of concentration or time limitations then the program will attempt to reconstruct a spectrum from the 2D NMR data. To establish the relative stereochemistry of the molecule either NOESY or ROESY spectral data are used.

In those cases when only 1D NMR spectra are available, the program performs a search in the Fragment Library based on the ^13^C NMR spectrum and, as a result, fragments whose subspectra are contained within the experimental spectrum are selected. Next, structure generation from the selected fragments is performed using overlapping common atoms to investigate the presence of larger fragments and, ultimately, to try and piece together a full structure solution. If this approach fails then the program automatically switches to the so-called "classical" generation mode, an approach which is common in the first-generation systems (e.g. [32]. This method has been described in detail in our previous work [35].

During the course of processing the 2D NMR spectral data the program analyzes the contour diagrams associated with the 2D spectra and determines the chemical shifts of the intervening nuclei (and therefore the coordinates of the peaks). The spectral parameters of the peaks are then imported into tables containing chemical shifts, intensities, and the multiplicities of the signals for the 1D spectra and the chemical shifts of the coupled nuclei and the intensities of the peaks in the 2D NMR spectra. It is also possible to input tables of 1D and 2D NMR spectrum peaks directly from a keyboard. Next, HMBC and COSY correlations are converted into connectivities represented by the chemical shifts of pairs of carbon atoms. Thus, for example, if a COSY spectrum exhibits a (H-*i*)↔(H-*k*) correlation then the connectivity involving the chemical shifts of the C-*i *and C-*k *atoms is produced.

##### Molecular Connectivity Diagram

**Figure 5:**
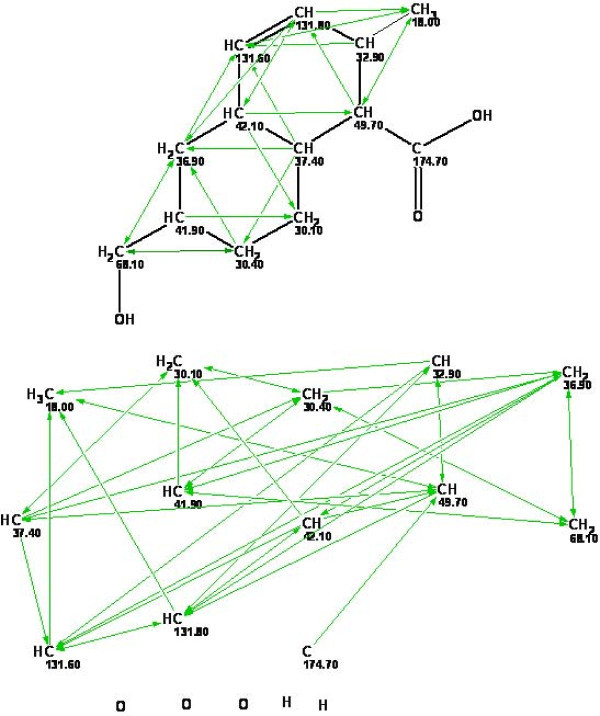
**Example of a structure and its Molecular Connectivity Diagram of HMBC connectivities**. In the structure, the HMBC connectivities are shown by arrows. It is seen that 131.6-18.0 and 36.9–131.8 connectivities are nonstandard (extending out above 3 bond correlations).

Further solution of the problem proceeds under the user's control in most cases. To provide a complete and clear pattern of the properties of the skeletal atoms and the connectivities between them the program places skeletal atoms together with hydrogen atoms attached to skeletal atoms in a display window. We refer to this visual depiction as a *Molecular Connectivity Diagram*, MCD (Fig. 5.). The values of the chemical shifts of the carbon and hydrogen atoms are accompanied with atom properties and are shown for each CH_*n *_group.

Obviously, if the hybridization state of the carbon atoms and the possibility of their bonding to heteroatoms are taken into account (i.e. specific constraints are introduced), then the process of structure generation is substantially accelerated. Therefore, with the use of the APCT library, the program sets (if it is possible) the most probable hybridization of each carbon atom (*sp*^3^, *sp*^2^, *sp*) and the possibility of that carbon being adjacent to a neighbor with heteroatoms ("forbidden," "at least one atom," "at least two atoms," "not defined"). The atom properties automatically assigned by the program can be edited by the user taking into account the chemical composition and additional information available from other spectral data (e.g., IR and Raman spectroscopy). If a distinct multiplet is observed in the ^1^H NMR spectrum from a structural block (C-*i*)H_n _then the total number of H atoms attached to carbons adjacent to the (C-*i*) carbon is set. This property is determined by the chemist after visual analysis of the ^1^H spectrum pattern and taking into account coupling constants (if measured). Note that the atom properties should be set and edited with great caution because an erroneous assumption leads to the exclusion of the correct structure from the output file. All structural constraints presented in the molecular connectivity diagram are used during the structure generation. In certain cases it is possible that one or more signals and too weak to be observed in the 13C NMR spectrum. This is especially common of course for quaternary carbon and carbonyl carbon atom. However, since the total number of carbon atoms is known from the molecular formula the absent carbon atoms are automatically included in the MCD and structure generation is then performed. In such cases the number of generated structures will be considerably greater because the inserted carbon atoms do not have their properties (hybridization, neighboring with heteroatoms, etc.) defined.

Before initiating structure generation the program automatically performs a logical analysis of the data presented in the MCD to check their consistency (i.e., the absence of nonstandard connectivities). Our algorithm proposed for the logical analysis and correction of the MCD is rather sophisticated and its complete description has been published previously [28]. When one or more nonstandard connectivities are found an attempt is made to resolve the contradiction automatically by the elongation of suspicious connectivities by one bond. This frequently allows structure generation from the corrected MCD. An attempt to perform structure generation without correcting the MCD leads to an empty output file or an invalid solution (i.e. the absence of the correct structure from the output file). The system includes approaches [27-30] for the detection of an invalid solution.

Our studies have demonstrated that in approximately 90% of all cases the program detects the presence of non-standard connectivities. However, even when the program yields a false conclusion that contradictions are absent, a valid solution can be obtained using *fuzzy structure generation*, which was first developed in our research [28,30]. This strategy uses approaches which provide a valid solution even at a very high degree of uncertainty of the initial information. The problem is formulated as follows: find a valid solution provided that the 2D NMR data involves an unknown number *m *(*m *= 1–15) of nonstandard connectivities and the length of each of them is also unknown. The efficiency of this proposed approach was verified by the examination of more than 100 real problems with initial data containing up to 15 nonstandard connectivities differing in length from the standard correlations by 1–3 bonds. Note that StrucEluc is the only system that includes mathematical algorithms for the search of contradictions and their elimination and, therefore, can work with real 2D NMR data.

##### Structure Generation and Verification

Structure generation is performed from the structural blocks C, CH, CH_2_, CH_3_, and heteroatoms when constraints are imposed that are entered as connectivities. Note that a group of carbon atoms showing COSY connectivities between them makes up a fragment (a connected subgraph), while each carbon atom connected to it via HMBC connectivities forms a "fuzzy fragment". Structure generation is controlled by options which impinge constraints on the sizes of the cycles and bond orders, introduce lists of *obligatory *and *forbidden *fragments and include a check for the fulfillment of Bredt's rule, etc. The use of the APCT library for setting the atom properties (see above) significantly accelerates the structure generation process. In particular, for 80% of ca. 300 problems that we have solved, the generation time was less than 1 min.

It is important to note that during the structure generation process chemical bonds are drawn between atoms possessing chemical shifts and properties specified in the MCD rather than abstract atoms. Therefore, in the generated structures, C and H atoms have assigned chemical shifts. The spectral filtration of structures proceeds simultaneously with generation, and three modes of filtration severity can be specified taking into account the ambiguity of boundaries of characteristic spectral ranges. It has been found that filtration in even the most relaxed mode leads to a decrease in the number of structures in the output file by a factor of tens and even hundreds [30].

##### Selection of the Most Probable Structure

For the correct elimination of duplicates and choice of the most probable structure the prediction of ^13^C NMR spectra and the calculation of the average deviations of the calculated spectra from the experimental data is used in the StrucEluc system. Therefore, ^13^C NMR spectra are predicted for all structures passed through the filters. Since an output file may be rather big (hundreds, thousand and even tens of thousands of structures) very fast algorithms for NMR spectrum prediction are necessary.

With this in mind we developed fast calculation algorithms [36-38], one of which is based on increments and the other employs artificial neural networks. These algorithms provide a calculation speed of 6000–10,000 chemical shifts per second with the average deviation of the calculated chemical shifts from the experimental shifts equal to *d *= 1.8 ppm. For a file containing tens of thousands of structural isomers the calculation time by the two methods is not longer than several minutes. Next, redundant isomorphous structures are removed. Since different deviations correspond to duplicate structures with different signal assignments the structure with the minimum deviation is retained from each subset of identical structures (i.e., the "best representatives" are selected from each family of identical structures). Isomers are then ranked by ascending deviation and our experiences show that the correct structure commonly is in first place with the minimal deviation or at least among the first several structures at the beginning of the list.

To check the correctness of the preferable structure the ^13^C and ^1^H spectra can be calculated for the first 20–50 structures from the ranked file with the use of the fragment method [23]. Although this method is not as fast as the other two methods it allows the user to obtain a detailed explanation how each predicted chemical shift was calculated. These calculations use a HOSE-code (Hierarchical Organization of Spherical Environments) based prediction approach (see review [22]) and employ a database containing 175,000 structures with assigned ^13^C and ^1^H chemical shifts. For each atom within the candidate structure, related structures used for the prediction can be shown with their assigned chemical shifts and this allows the user to understand the origin of the predicted chemical shifts. Finally, the file is repeatedly ranked by ascending *d*_*A*_, where *d*_*A *_is the deviation of the calculated ^13^C spectra using the fragment method. If the difference between the deviations calculated for the first and second ranked structures is small [*d*(2) - *d*(1) < 0.2 ppm] then the final determination of the preferable structure is performed by the expert. Generally the choice is reduced to between two or, less frequently, three structures. In difficult cases, the ^1^H NMR spectra can be calculated for a detailed comparison of the signal positions and multiplicities in the calculated and experimental spectra. Solutions that may be invalid are revealed by a large deviation of the calculated ^13^C spectrum from the experimental for the first structure of the ranked file. For instance, if *d*_*A*_(1) > 3–4 ppm the solution should be checked using fuzzy structure generation. The reduced *d*_*A*_(1) value found as a result of fuzzy structure generation should be considered as a hint regarding the presence of one or more nonstandard connectivities. The correct solution is usually obtained using different modes of fuzzy structure generation [30]. The NOESY spectrum [39], which imposes constraints on geometric distances between intervening protons can also give valuable structural information (spatial constraints) at this step.

In the course of structure elucidation of natural products we encountered a number of unknown molecules with symmetry. The presence or absence of molecular symmetry does not influence structure generation within 1D expert systems. We determined that an attempt to generate symmetric structures from 2D NMR data lead to unmanageable processing times. The cause of this failure was carefully investigated and peculiarities of symmetric structure generation were discovered. An enhanced algorithm was developed to reveal symmetry features in 2D NMR data and then it is automatically adjusted to the generation of symmetric molecules. As a result the processing time necessary for the generation of symmetric molecules is now of the same order as for molecules without symmetry. This algorithm is still not optimized and we are presently researching improvements to allow further acceleration of the structure generation process by using symmetry for this purpose.

From the very beginning the StrucEluc system was intended for the identification of standard organic molecules. Since ionic structures are possible for both natural products and synthesized organic molecules the structure generation algorithm was also adjusted to allow for the generation of ionic structures. Thus, in principle the system is now capable of elucidating structures of any organic molecules regardless of their topological and electronic properties.

Let us consider two examples demonstrating the application of the StrucEluc system in the Common Mode.

##### Examples of problem solving in Common Mode

###### Problem solution using Crisp Structure Generation

**Figure 6:**
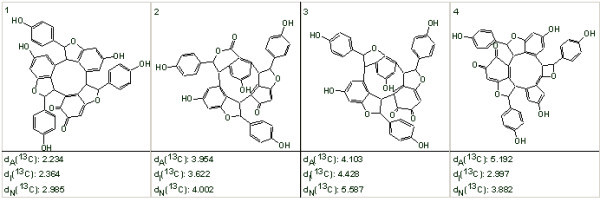
**The four structures at the top of the ranked structural file**. The first ranked structure is identical to the structure of *Hopeanolin *determined by authors [40].

Ge *et al *[40] isolated and determined the structure of a new unusual natural product named *Hopeanolin*. To challenge StrucEluc we used published 1D and 2D NMR data [40] to elucidate the "unknown" structure. 80 HMBC and 3 COSY correlations were input into the program and the MCD was created. Atom hybridization was automatically set for all carbons except 8 CH atoms and two quaternary C atoms with chemical shifts in the range 90–120 ppm. In so doing the program took into account that chemical shifts observed in this region can be assigned either to C = C or to O-C (O-C-O) carbons. A single obvious constraint was imposed: three sp^2 ^carbon atoms with chemical shifts between 170 and 180 ppm were marked as having two neighboring oxygens each. No nonstandard correlations were detected in the 2D NMR data by checking the MCD. The results of the structure generation and filtering were: 68 structures were generated in 12 s, and 9 structures were stored after filtering (we denote this as *k *= 68→9). ^13^C NMR chemical shifts were predicted for all structures using all of the fragment, incremental and neural net approaches. The four structures at the top of the ranked structural file are shown in Fig. 6., where the "best" structure is *Hopeanolin*.

###### Application of Fuzzy Structure Generation

In the analysis of *cleospinol A *[41] with molecular formula C_20_H_32_O_2_, the 2D NMR data are comprised of 21 COSY and 55 HMBC correlations. These data were used to evaluate the possibility of solving a problem in those cases when a large number of nonstandard correlations were present. In this case the 2D NMR data contained 3 HMBC and 12 COSY nonstandard correlations whose length should be enlarged by 1–3 bonds.

**Figure 7:**
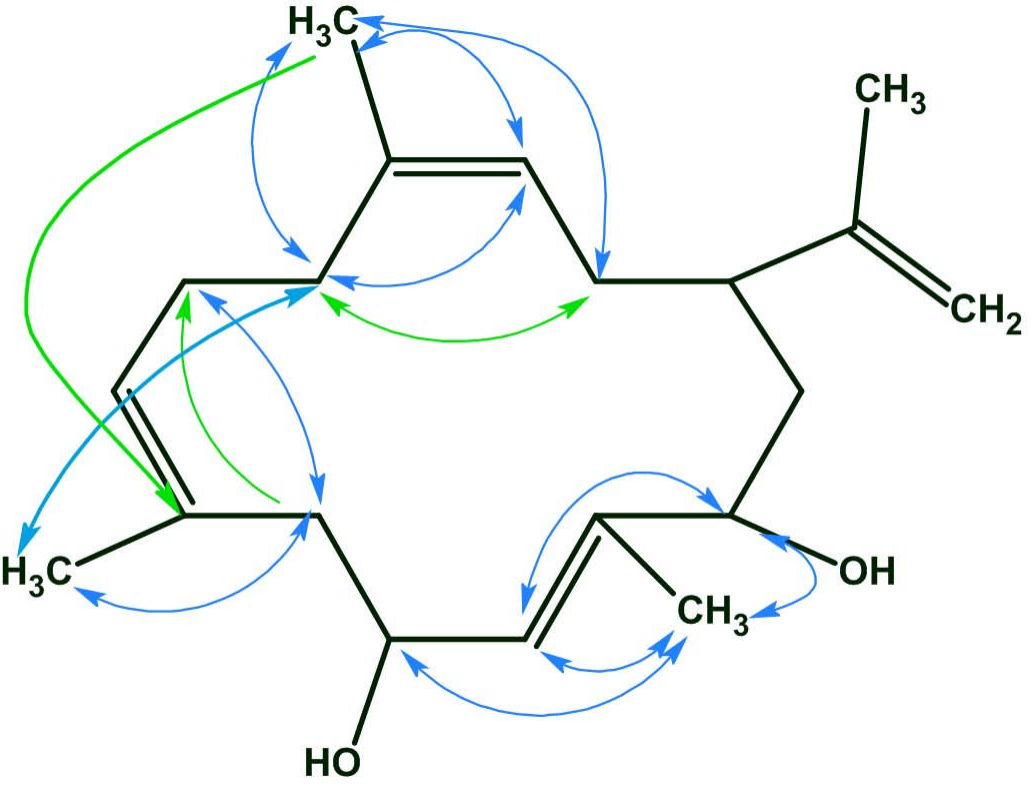
**The structure of *cleospinol A*. COSY correlations are denoted by blue arrows, HMBC correlations – by green arrows**.

As shown in Fig. 7. the COSY connectivities are represented below on the structure by blue double-headed arrows while the HMBC correlations are defined by green unidirectional arrows from the proton to the carbon to which it is long-range coupled.

The COSY, HMQC, and HMBC spectral data associated with the compound were fed to the program and the MCD was generated. A check of the MCD was accompanied by the automatic removal of contradictions. The software program displayed a message declaring that contradictions had been detected and resolved while the minimum number of NSCs was estimated to be equal to 7. Unfortunately, strict structure generation from the automatically edited MCD resulted in an empty output file. This result was interpreted as evidence of the presence of either undetected additional nonstandard correlations or those whose lengths must be augmented by more than one bond. Detailed analysis of this problem and possible ways of its solution are reported elsewhere [30]. Here we will describe the most universal and systematic approach. Fuzzy structure generation was initiated assuming only that the number of nonstandard connectivities numbered no more than 15. In this case 18,281,379 connectivity combinations from 40,225,345,056 theoretically possible combinations were used for structure generation. The following result was obtained: *k *= 769→430→245 (245 structures retained after removing duplicates), a generation time of *t*_*g *_= 29 min 9 sec, and the correct structure was ranked first by all methods of spectrum prediction.

The program therefore was able to identify the correct solution even when 15 nonstandard connectivities existed in the 2D NMR data and especially in the presence of HMBC and COSY connectivities representing both ^6^J_CH _and ^6^J_HH _correlations. Note that only ~10^-4 ^of the theoretically possible connectivity combinations were processed. The real number of processed connectivity combinations *n*_*real *_was > 18 million. Nevertheless, the high-speed structure generator present in the Structure Elucidator program completed the process in a reasonable time.

#### The Fragment Mode of Operation

As shown above computer-assisted structure elucidation using 2D NMR data is quite efficient for the determination of structures of complex natural compounds. However, if the structural restrictions imposed by the MCD are not sufficient for the generation of a reasonable number of possible structures within an appropriate time, it is to be expected that the utilization of molecular fragments can greatly facilitate the solution of the problem. The *fragment approach *has been successfully used in all first generation expert systems based on 1D NMR spectra only. However in those cases when 2D NMR data are employed, the utilization of molecular fragments is hampered because *all *carbon atoms existing in a fragment used in solving the problem *must *be supplied with chemical shifts. Moreover, the values of these chemical shifts must be as close as possible to the observed values for the atoms of the corresponding fragments in the experimental ^13^C NMR spectrum of the unknown under study. In addition, the accommodation of one or more fragments within a set of connectivities derived from the 2D NMR data is a problem that required the development of new algorithms. Appropriate fragments to aid in the solution of a problem can usually be found in the fragment library of the StrucEluc system. The main advantage of these fragments is that all fragment carbon atoms are supplied with the ^13^C NMR assignments obtained from the full structures that were used for creation of the fragment data base.

The first step in the process is a fragment search of the fragment library (FL). As a result, a set of ***L ***found fragments is selected. The next step is the creation of the MCDs using the found fragments (FF). For this purpose, either all FFs or any selected number of them can be incorporated by the operator. An algorithm that implements this procedure was developed and realized within StrucEluc system [25]. The program produces all rearrangements of the experimental chemical shifts within the corresponding carbon atoms of the fragment. The chemical shift distribution of carbon atoms that produces a conceivable assignment of a given fragment has to be verified. During the verification process, the program checks whether or not the carbon atom assignments correspond to the experimental chemical shift *correlations *comprising the skeletal atoms making up the fragment. The fragments that survive the test are then included in the set of *prospective *fragments.

The more skeletal atoms that are accounted for by the fragments then the shorter the process of structure elucidation. With this in mind an algorithm combining the prospective fragments within one molecular connectivity diagram was developed. To realize this procedure, all possible combinations of prospective fragments are searched and only combinations that are in agreement with the experimental 2D NMR correlations are chosen. The fragment combinations that pass this examination form a set of *prospective fragment combinations*. These fragments are then "projected" onto the MCDs together with any remaining free atoms. The user can then visually analyze these MCD diagrams.

The speed of structure generation depends on the size of the molecular fragments. If the number of small fragments composing the MCD is large enough then this will speed up the generation process. Structure generation is also much faster when the MCD is comprised of only a small number of large fragments. Depending on the size of the molecule being analyzed and the size of fragments placed at the beginning of the ranked list of found fragments, the number of fragments included into the MCD usually varies from 1 to 4.

The conclusion of all further verification procedures is a check of all of the MCDs produced for the presence of contradictions. The program offers an option that deletes all MCDs that are recognized as containing contradictions. The contradictory MCDs contain fragments with carbon atoms associated with assignments that contradict the standard length of the corresponding connectivities. The diagrams remaining after checking can be used in the structure generation process.

In the process of analyzing a novel compound, it is entirely possible that there will be no fragments in the database that will reduce the magnitude of the challenge. It is natural in such cases to expect that the introduction of user-defined fragments may help to form the MCDs. The main qualitative difference between a found fragment (FF), and a user fragment (UF), is that the FF already contains carbon atoms with assigned chemical shifts while the carbon atoms of the UF have no carbon chemical shift assignments. Two ways have been suggested to introduce user fragments into the program:

• calculate the carbon chemical shifts of the fragment;

• search the FL for fragments that *comprise *the user fragment.

It is likely that fragments from at least one of these two sources will be available for use by the program. Experience has shown [25-27] that an appropriate combination of FFs and UFs frequently allows the solution of rather difficult problems.

##### Example

**Figure 8:**
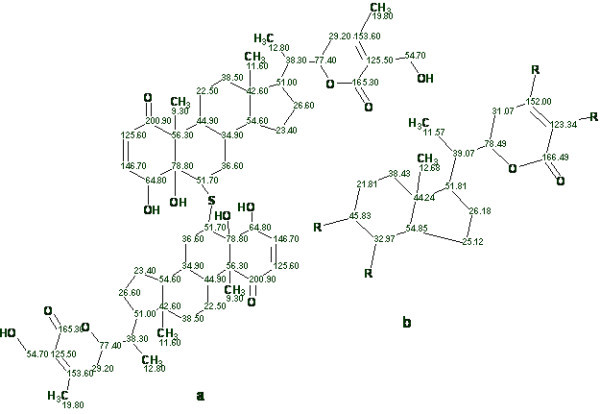
**The structure of *ashwagandhanolide *(a) and a Found Fragment (b)**.

A new dimeric natural product, *ashwagandhanolide *(Fig. 8a), was isolated by Subbaraju *et al *[42]. Its molecular formula was determined as C_56_H_78_O_12_S on the basis of the molecular ion observed at *m*/*z *975.5285. The structure of this compound was determined using 2D NMR data as well as additional information obtained from comparison of experimental spectra with the structures and spectra of related molecules. In the article [42] only 35 HMBC correlations are reported (there is no COSY data). The number of correlations is small due to severe overlap in the ^1^H NMR spectrum. An attempt to solve the problem using StrucEluc in Common Mode showed that the processing time and the number of generated structures would be unmanageable. Therefore a fragment search using the ^13^C NMR spectrum was performed and 5524 fragments were found in the Fragment Library. The displayed Found Fragments are ranked in decreasing order of the number of carbon atoms, and the first ranked fragment is shown in Fig. 8b. Visual comparison of the molecular structure with the structure of the fragment confirms that the fragment is a substructure of the structure and its carbon chemical shifts are very close to the values measured for the full structure. The procedure of creating MCDs from the Found Fragments was initiated and the program produced 960 MCDs with different shift assignments. Checking MCDs for contradictions took 28 min, and structure generation resulted in k = 960→24→6, *t*_*g *_= 22 s.

**Figure 9:**
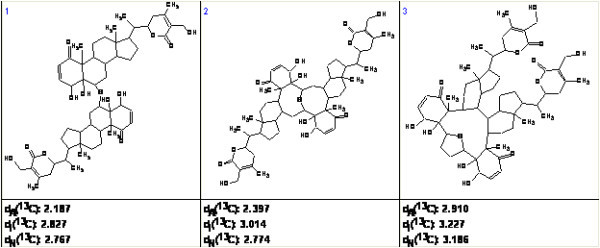
**The three top structures of the ranked file**.

The three top structures in the ranked file are shown in Fig. 9. The most probable structure 1 coincides with the structure determined in the publication [42].

#### User Database application

Currently, about 27 million compounds have been identified and well over a quarter million new compounds are synthesized or isolated each year. It is possible that many such compounds will have no analogues in the knowledgebases of expert systems. As a result it is not always possible to find fragments in the database that will help to elucidate the structure of a compound from a new class of chemicals. Besides, it is common that an investigator is unable to specify a fragment that may be present in the structure under examination. Investigation [25,27,43,44] has shown that if the methods described above are ineffective, then the creation of a user database could enable a solution. The *StrucEluc *system provides algorithms and capabilities to create user databases and thereby to allow searches for fragments of related compounds. In particular, even if only one compound of a similar structure is known, it can be successfully used for the creation of a user database. With the help of user databases the system can easily be adjusted for the elucidation of compound classes that are commonly investigated by a given laboratory. Investigations have shown that the failure to utilize library fragments was most frequently due to the following issues: a) the fragments appropriate for a given problem are missing in the knowledgebase; b) appropriate fragments are found but the number of possible permutations of the carbon atom assignments in these fragments is so huge that the structure generation process is too long; c) the molecule under investigation is characterized by a deficit of hydrogen atoms and as a result the 2D NMR correlations do not deliver a number of constraints enough to produce a manageable output file in a reasonable time. As an example we will consider utilization of the user database for the structure elucidation of natural products of the cryptolepine series.

**Figure 10:**
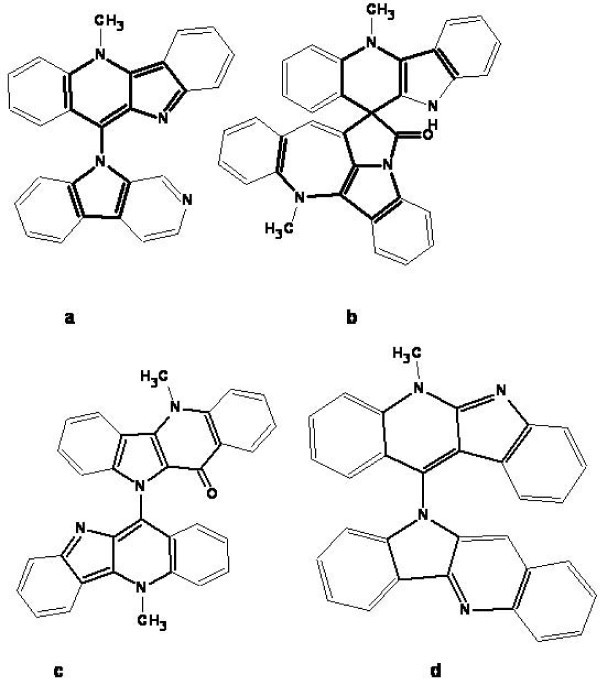
**Examples of structures of the natural products which failed to be elucidated both in the Common and Found Fragment modes**.

The structures of the natural products which failed to be elucidated both in the common and found fragment modes are shown in Fig. 10.

These molecules are relatively large, highly unsaturated, and have large "silent" fragments (displayed in bold) that contain no hydrogen atoms, thereby preventing access to structural information through COSY correlations. In particular, for structures **a-d **COSY correlations are observed for protons contained within the benzene rings only. These factors contribute to a very challenging elucidation process for these compounds.

**Figure 11:**
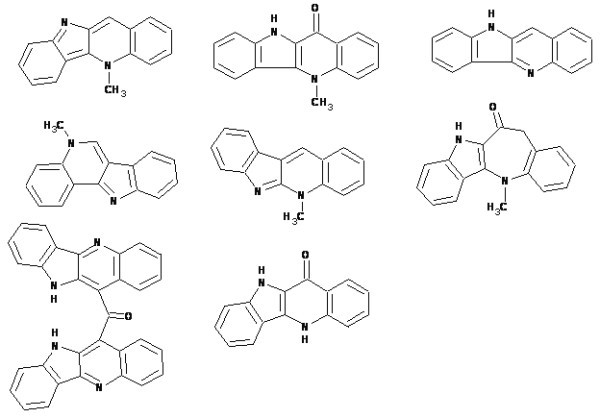
**Compounds of the cryptolepine series used to create User fragment library**.

The elucidation of the structures of alkaloids **a-c **was approached using a user database adjusted for cryptolepine structure analysis. The main assumption was that unknown compounds were members of the cryptolepine indoloquinoline alkaloid series since all materials were isolated from the same plant. Taking this into account, information regarding earlier published members of the series shown in Fig. 11 was introduced into a user database. Assigned spectral data referring to these compounds were obtained from original publications. This methodology is fairly typical in practice and is frequently used by chemists when manually determining the chemical structures of natural products or reaction products from a synthesis.

Compounds of the cryptolepine series were incorporated in the user library as full structures along with their associated ^13^C NMR spectra. The algorithm used to create a User Fragment Library is similar to the algorithm used to create the *StrucEluc *system knowledgebase and includes the following two basic steps:

1) The program excises as complete as possible a set of fragments from all structures included in the structural file. 2) Atoms in the fragments are assigned chemical shift values that they have in the corresponding full structure. This procedure produced a user library containing 342 fragments from the cryptolepine series. This user database was then successfully used to elucidate structures **a-c**. The elucidation process is described in details in our other works [25,27,43].

This approach has been also applied to the solution of a particularly challenging problem that remained unresolved for over a decade [44]. The structure of an unknown alkaloid **d **(Fig. 10), quindolinocryptotackieine, also belonging to the cryptolepine family, was elucidated. NMR data were collected starting in 1991 and a solution remained elusive, in part due to extensive spectral overlap in both the ^1^H and ^13^C spectra. Manual analysis did not present a conclusive structure while computer-assisted elucidation produced a series of conceivable structures. A single structure consistent with all of the spectral data was selected from the list of computer-generated structures. To the best of our knowledge, this is the first case of the application of an expert system to determine the molecular structure of a complex natural product which was not amenable to identification by competent spectroscopists.

### Impact of rapid NMR spectrum prediction on system properties and capabilities

Traditionally developers of expert systems aspired to use all possibilities to provide a solution to a problem as a minimum structural file satisfying all the constraints imposed by spectra and *a priori *information. The shorter the list of candidate structures the simpler is the choice of the most probable structure. The crucial constraint is the processor time necessary for NMR spectrum prediction for all structures contained within the output file. From the general considerations explained in the Section 2, it is clear that the output file can be markedly reduced by imposing severe constraints ("axioms") capable of rejecting as many as possible conceivable isomers. Application of severe constraints does however increase the risk that the genuine structure will be lost and the solution will become invalid.

The situation improved when new incremental and artificial neural net methods of ^13^C chemical shift calculation were produced [36-38]. High speed chemical shift prediction (6000–10,000 shifts/s) now allows calculations to be generated for a file containing hundreds of thousands of structures in 5–10 min and as a result there is no necessity for imposing the strict constraints. The refusal of too rigid and risky constraints sufficiently raises the reliability of solutions obtained with the assistance of the StrucEluc system.

#### Accelerating the Structure Generation Process

The Structure Generation algorithm is arranged in such a way that first it produces substructures which are then complemented by new bonds until full structures are generated. It is possible to consider that structures generated from a given substructure make up a definite branch of the whole generation process. If the "root" substructure contains a carbon atom C-*i *with such an environment for which the predicted chemical shift δ(C-*i*) contradicts the experimental ^13^C NMR spectrum, the whole structural branch will be truncated during the spectral filtering process. We believed that fast ^13^C chemical shift prediction for incomplete structures during the generation process would prevent generation of such structural branches that deliberately contradict the experimental ^13^C NMR spectrum. With this in mind we developed a pilot version of a structure generator that is enhanced by the increment based ^13^C chemical shift calculation of incomplete structures. The main problem that should be resolved is to find a compromise between the number of HOSE code environment spheres taken into account during the chemical shift calculation for a given carbon atom and balancing it with saving structure generation time. This algorithm is rather complicated and work is presently underway to improve the system. To date the coefficient of acceleration varies between 1 and 70 depending on the nature of the problem. The coefficient is expected to be greater for molecules containing many heteroatoms and different functionalities.

#### Rapid verification of structural hypotheses

As shown above the StrucEluc system is capable of inferring all conceivable structures that agree with the experimental spectra and the additional constraints imposed by the chemist. NMR spectrum prediction is then used to select the most probable structure and compare it with other structures ranked in decreasing order of probability. The advantages of this systematic approach are obvious: all structures are exhaustively enumerated, ranked and automatically displayed.

The overwhelming majority of structures published in the literature were elucidated by traditional methods using knowledge, experience and the intuition of organic chemists. Obviously the human expert is frequently unable to derive and check all possible structures. Therefore, it is not surprising that sometimes incorrect structures are elucidated by chemists. A recent example is the high-profile case of the hexacyclinol structure (C_23_H_28_O_7_). Schlegel *et al *[45] separated a natural product and suggested its structure based on the 2D NMR data. Le Clair [46] reported a synthesis of the structure, but Rychnovsky [47] suggested another structure based on ^13^C chemical shift prediction using a GIAO-based quantum-mechanical DFT (Density Functional Theory) prediction method. He showed that the calculated spectrum of his structure was more consistent with the experimental spectrum than the calculated spectrum of Schlegel's structure. Rychnovsky's suggestion was confirmed by Porco *et al *[48] who synthesized the suggested structure and unambiguously determined the structure using X-rays. It was later shown [49] that if StrucEluc was used for the structure elucidation of this particular natural product the researchers would have easily avoided their erroneous suggestions.

Rychnovsky convincingly showed that quantum-mechanical DFT chemical shift calculations could be very useful and efficient for the structure elucidation of complex organic molecules in spite of significant time investments (the total processing time for an isomer C_23_H_28_O_7_was approximately 12 h using a 3 GHz Pentium 4 processor based computer).

A series of articles have been published (for instance [50-52]) where DFT NMR spectrum prediction was successfully used for this purpose. However as mentioned earlier quantum-mechanical calculations are time consuming and require a knowledgeable and skilled expert to obtain reliable results. We suggested that the application of our fast and accurate chemical shift prediction algorithms could help in many cases without the need for quantum-mechanical calculations or even in their place. Our suggestion was confirmed by empirical NMR spectrum predictions performed for many structures taken from the literature for which the DFT approach was used to verify different structural hypotheses. We recently submitted an article describing a series of interesting cases [53]. We consider below two examples applying the StrucEluc application to the revision of structures

**Figure 12:**
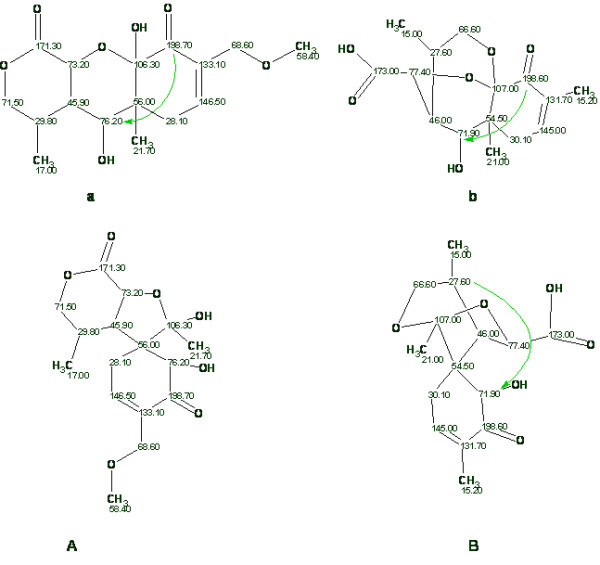
**The originally proposed (*a*, *b*) and revised structures of *Boletunone A *and *B***.

Kim *et al *[54] described the structures and properties of two new natural products and attributed them to *Boletunone A *and *Boletunone B *(see structures ***a ***and ***b ***in Fig. 12). Steglich and Hellwig [55] have shown that both structures suggested by the authors [54] are wrong, and alternative structural formulae (**A **and **B**, Fig. 12) were offered and proved for these compounds.

We have investigated the potential of using StrucEluc to analyze the data of Kim *et al *and both structures have been elucidated in a systematic manner.

##### Boletunone A

**Figure 13:**
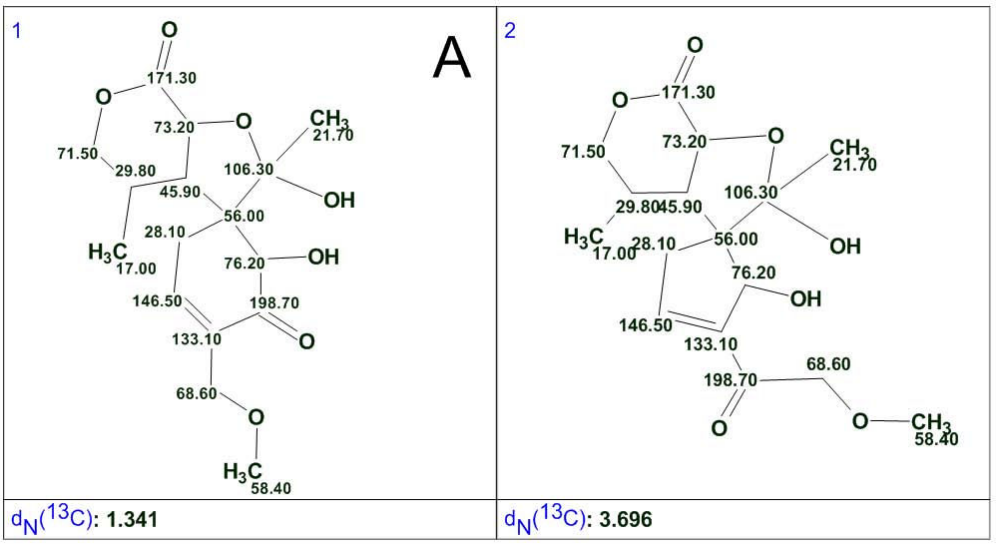
**Solution to the problem for *boletunone A***.

As shown in Fig. 12. in order to accept the proposed structure ***a***, the authors [54] postulated that the length of one HMBC correlation (198.7→76.2) corresponds to ^4^J_CH_. When the 1D and 2D NMR spectra (COSY and HMBC) were input into the StrucEluc program the first run was performed in Common Mode with crisp Structure Generation. The two structures presented in Fig. 13. were generated in 0.15 s and the ^13^C chemical shifts predicted by neural nets, and the average deviations *d*_*N*_(^13^C) are shown.

**Figure 14:**
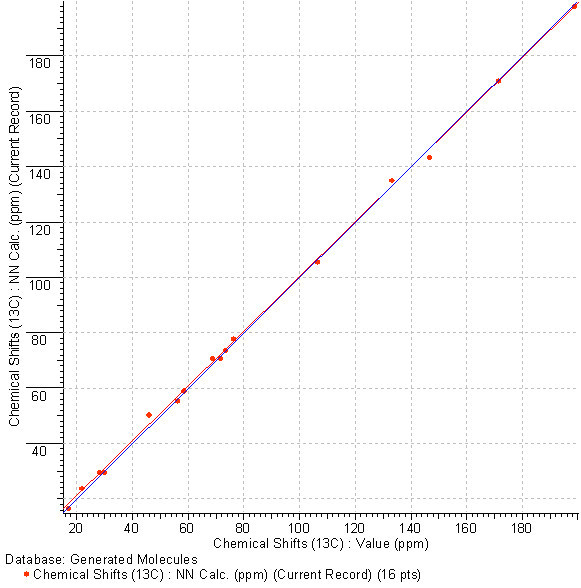
**The correlation between calculated and experimental ^13^C NMR spectra for structure A**. The target line Y = X is colored in blue.

The revised structure, **A**, was unambiguously distinguished as the best structure by the average chemical shift deviation, while structure ***a ***was not generated at all because the presence of nonstandard correlations was not allowed. Note that the quality of spectrum prediction for structure **A **is characterized by R^2 ^= 0.999 and a graph of the linear regression shown in Fig. 14. demonstrates almost perfect coincidence with the target line Y = X.

As a next step Kim's declaration regarding one non-standard correlation was taken into account and Fuzzy Structure Generation was initiated under a condition that 3- and 4-membered cycles are forbidden.

Result: *k *= 18→13→10, *t*_*g *_= 0.7 s.

**Figure 15:**
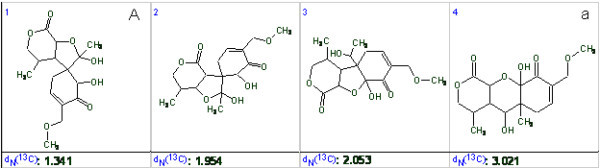
**The top of the file containing structures ranked by *d*_*N*_(^13^C) values**. The revised structure **A **is in first position while the structure ***a ***is placed in fourth position.

The first four structures of the ranked output file are shown in Fig. 15.

**Figure 16:**
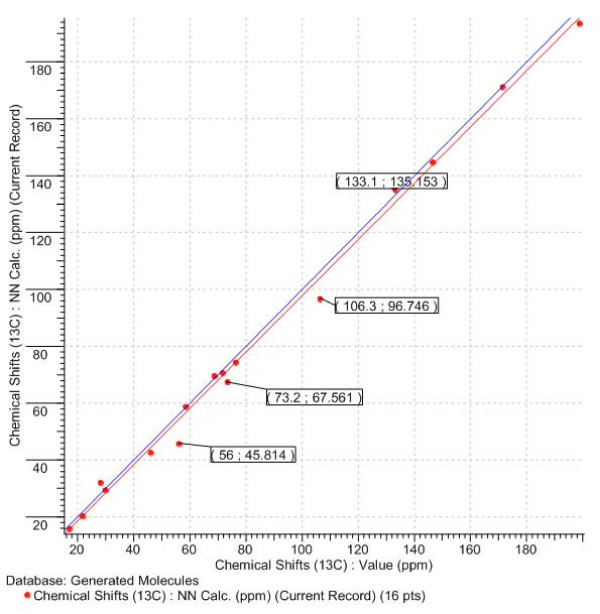
**The correlation between calculated and experimental ^13^C NMR spectra for structure a**. The target line Y = X is colored in blue. The first number in the frames denotes the experimental value of a chemical shift and the second number represent the calculated one.

The correct structure **A **was again unambiguously determined while the original structure ***a ***was ranked in fourth position with a *d*_*N*_(^13^C) value more than twice as large than for the first-ranked structure. The R^2 ^value extracted for structure ***a ***is equal to 0.995 but the regression graph (Fig. 16.) demonstrates significant scattering of the calculated shifts.

##### Boletunone B

Bagno *et al *[50] have used DFT chemical shift prediction to validate structure **b **of *Boletunone B *[54] in comparison with the revised structure **B **proposed in work [55]. They calculated the ^1^H and ^13^C spectra both for the original and revised structures. The geometries were optimized at the B3LYP/6-31G(d,p) level in the gas phase, and the NMR properties calculated with B3LYP/cc-pVTZ, in the gas phase and with a solvent reaction field of DMSO. The mean absolute errors were corrected to take into account the systematic errors of the DFT chemical shift calculations.

The authors [50] showed that the ^13^C spectrum calculated for the revised structure **B **is in better agreement with the experimental spectrum than the spectrum calculated for the originally proposed structure **b**.

The 1D and 2D NMR data of *Boletunone B *presented by Kim *et al *[54] were input to StrucEluc and Crisp Generation was performed – no nonstandard correlations were assumed in the 2D NMR data for the first run. The results gave: *k *= 4343→37→31, *t*_*g *_= 12 s.

**Figure 17:**
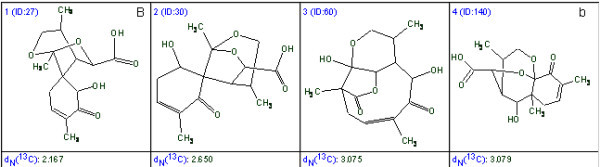
**The beginning of the file containing structures ranked by *d*_*N*_(^13^C) values**. The revised structure **B **is ranked in first place while the incorrect structure ***b ***is placed in fourth position.

It turned out that neither structure ***b ***nor structure **B **were generated, while the "best" structure in the ranked structural file had a *d*_*N*_(^13^C) value equal to 3.1 ppm. The result obtained hints that at least one nonstandard connectivity is present in the 2D NMR data (Kim *et al *[54] assumed one NSC in HMBC data). Structure generation was repeated in the Fuzzy Mode and the presence of one NCS was allowed in the HMBC data only. The results gave: *k *= 37176→149→135, *t*_*g *_= 1 m 40 s. The top ranked structures are shown in Fig. 17.

The revised and correct structure was again recognized as the most probable using neural net based ^13^C chemical shift calculation, while the original structure ***b ***was ranked only in fourth position. The values of R^2 ^calculated for structures **B **and **b **are equal to 0.999 and 0.995 while the maximum chemical shift deviations are 3 ppm (**B**) and 9 ppm (***b***).

These examples show that the application of a systematic approach that was thoroughly developed and tested by solving hundreds of problems in combination with new fast and accurate algorithms of NMR spectrum prediction gives correct solutions very quickly and without the application of cumbersome and time-consuming quantum-mechanical methods. When several structural hypotheses should be compared prior to applying DFT methods it is worth validating competing structures using more generic NMR predictors such as those discussed in this work. Quantum-mechanical calculations can play a decisive role in selecting the right structure if empirical NMR spectrum prediction fails to calculate the chemical shifts of some carbon atoms in an unusual environment not described in the training or reference structural sets. DFT calculation may also help when the average deviations calculated for the competing structures are very close and there is no reliable basis for making the final decision.

### The determination of relative stereochemistry of elucidated structures

The biological activity of natural products and drug molecules can be highly dependent on the stereochemistry of a molecule. Indeed, there are examples known where one stereoisomer can exhibit vastly different pharmacologic activity from the other stereoisomer. As an example, D-propoxyphene has analgesic activity while the other optical isomer, L-propoxyphene, has antihistaminic activity. Stereoisomer considerations can influence reaction pathways and certainly reaction kinetics, with one form being favored over another. Generally, the final step in contemporary structure characterization efforts is to define the relative and if possible absolute stereochemistry. NMR methods are generally well suited to the former while the latter is generally obtained using chemical structure modification combined with NMR studies or by X-ray crystallographic methods. NMR-based determination of relative stereochemistry is based on the nuclear Overhauser effect (NOE), which is dependant on the distance separating the cross-relaxing nuclides [39]. Typically NOESY or ROESY two-dimensional NMR experiments or their selective 1D analogs are used to provide the data for this analysis in rigid molecules.

The determination of the relative stereochemistry for new organic compounds, especially natural products, has become a routine procedure which needs to be speeded up and automated as much as possible. Conditionally, it is conceivable to distinguish the following two stages in the traditional strategy of relative stereochemistry determination: 1) selection of a set of the most probable stereoisomers using similar reference structures for which the relative stereochemistry has been determined; 2) examining these stereoisomers by NOESY/ROESY spectra, molecular modeling and quantum-chemical ^13^C NMR prediction for finding the most preferable stereoisomer. Methods allowing computational modeling of stereochemistry have been developed and incorporated into StrucEluc system.

#### The selection of the set of the most probable stereoisomers

When ACD/NMR predictors were developed information regarding the relative stereochemistry of reference molecules was taken into account. As a result, NMR spectra were calculated by all three methods (fragmental, incremental and neural nets) and these computational approaches are sensitive to different degrees to the orientation of the stereobonds in the structure under investigation. We undertook a study aimed to evaluate the possibility of using empirical NMR chemical shift prediction as a tool to select the set of most probable stereoisomers [56]. We found that the fragmental approach was the most sensitive to stereochemistry of the three methods and showed that it can be used for the purpose of stereochemical investigations. The approach was examined using a series of new natural products reported in the literature in 2008 and belonging to a number of different classes – steroids, alkaloids, terpenes, cembranoids, etc. The stereochemistry of the compounds was reported in the corresponding publications. The structural formula of each structure examined was input into the Proposed Structure window of StrucEluc, and all N = 2^n ^(n – number of stereocenters) mathematically conceivable stereoisomers were generated and depicted by the program. In our experiments the value of N varied between 64 and 4096 (n = 6÷12). ^13^C NMR chemical shift prediction was then performed and the stereoisomers were ranked in descending order of the average deviation between the experimental and calculated spectra. The study showed that the correct stereoisomer is usually placed at the top of the ranked file and took between 1^st ^to 3^rd ^position in the list therefore allowing the program to serve as a filter capable of rejecting non-perspective stereoisomers. Note that NOE data were not even used at this stage. Subsequent visualization of the NOESY/ROESY connectivities on the structures allows rapid determination of the most preferred member of the "best" isomers set.

**Figure 18:**
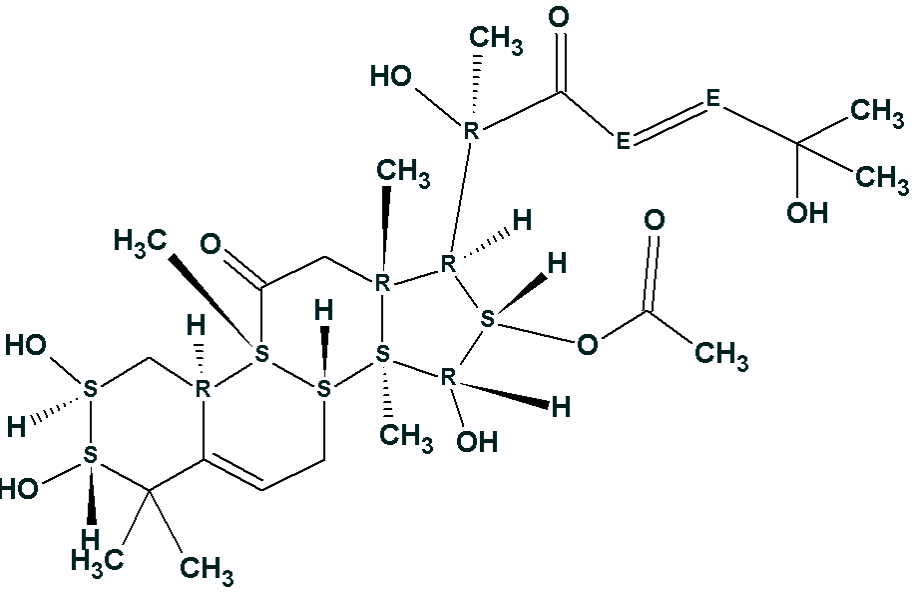
**Structure of the cucurbitacin**. Stereocenters are automatically labeled by the program.

**Figure 19:**
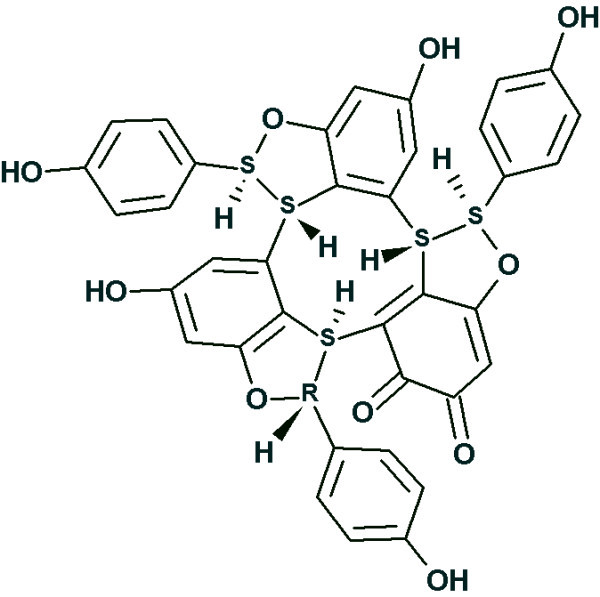
**Structure of the right stereoisomer**.

For example, Maloney *et al*. [57] reported the structural characterization of two new cucurbitacins, one of which is shown in Fig. 18 (12 stereogenic centers were determined and marked by the StrucEluc program automatically):

^13^C NMR spectra were calculated for 4096 stereoisomers in ca. 3 h and the ranking procedure promoted the correct structure to the first position. Note that spectra could be calculated only for 2048 stereoisomers due to presence of enatiomeric pairs which have identical spectra (the program adjusts to this peculiarity of the full stereoisomer family). In another example, for the structure elucidated and reported in section *3.2.1.1 *the correct stereoisomer (among 64 possible structures, n = 6) was placed by the program in third position in the rankings (Fig. 19):

Nevertheless, the final determination of relative stereochemistry requires additional confirmation. In publications where DFT based ^13^C chemical shift prediction was used for selection of the most probable stereoisomer of natural products, the set of stereoisomers to be examined was preliminary distinguished by the user on the basis of different experimental data. We hope that the described approach can ease selection of the set of stereoisomers to be tested by QM spectral prediction and reduce the costs associated with time consuming GIAO based calculations. Empirical methods are expected to be more reliable when the structure under investigation is relatively rigid.

#### Simultaneous determination of 3D model and relative stereochemistry

The StrucEluc system was also enhanced by adding an algorithm to allow the determination of 3D model and the relative stereochemistry of a molecular structure based on nuclear Overhauser effect constraints. The program extracts NOE information from either NOESY and/or ROESY spectra and determines the molecular stereochemistry accordingly. Results of selective NOE or ROE experiments can also be used as input to the program. This process can be carried out for several of the most likely structures produced during a structure elucidation by the expert system or performed on a chemical structure proposed by the chemist.

The utility of NOESY/ROESY spectra for relative stereochemistry determination is based on a direct correlation between both the crosspeak volume integration and the internuclear distance. Peak intensity in NOE/ROE measurements has an inverse sixth power relationship. Consequently [58], what can be referred to as a "strong" NOE is generally observed between pairs of hydrogens which are 1.8–2.5 Å apart. Responses of "medium" intensity usually correspond to an internuclear distance of 2.5–4.0 Å while "weak" NOEs will generally be observed for larger distances if they are observed at all. NOE responses are not commonly observed for nuclei farther than 5.0–6.0 Å apart. These data can be used to minimize energy to obtain the optimal molecular geometry.

Minimization algorithms deal with *numerical values *and, in this case, these numerical values are extracted from a set of NOEs overlaid on a 3D structure and examined for goodness of fit. The function describing this goodness of fit is called a *penalty function*. The better the solution then the lower the value of the function. The function must exhibit the lowest value for the best-matching stereoisomer.

In our work [59], an appropriate function was suggested that could be minimized by calculation for *all *stereoisomeric structures or by using a stochastic genetic algorithm to limit the number of stereoisomers that need to be investigated [60]. To improve genetic algorithm convergence, efficient methods of parameter optimization were suggested and compared. Since the chromosomes used here only incorporate information about stereocenter configuration, and not information such as chair or boat ring conformations, conformational rigidity is essential to obtain accurate results. This requirement presently limits the algorithm to the fused ring portions of molecules. Meanwhile relatively simple problems of 2–6 chiral centers may be solved in a straightforward manner by the enumeration of all stereoisomers and the calculation of the corresponding target function values. This process is fast and this approach is preferred over the use of genetic algorithms for molecules with smaller numbers of stereocenters.

**Figure 20:**
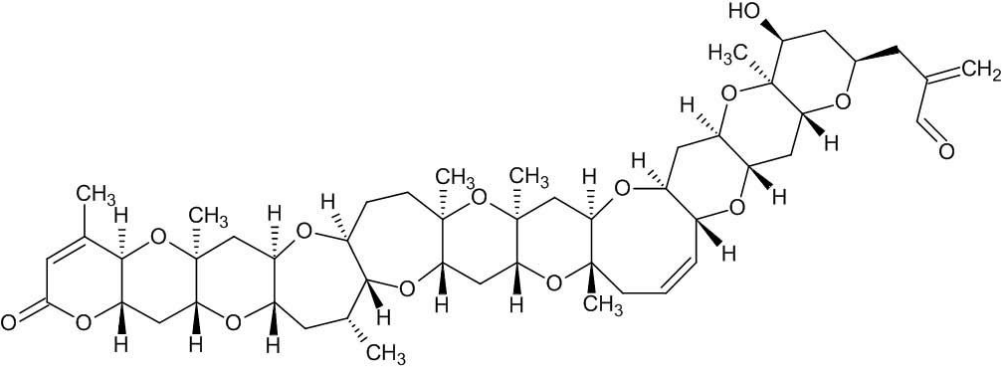
**The structure of brevetoxin B**. The configurations of stereocenters are determined by X-ray analysis.

A method for using a stochastic genetic algorithm for the elucidation of the stereochemistry and determination of the 3D geometries of potential molecular structures was demonstrated by the application of the suggested approach to two complex natural products Taxol (C_47_H_51_NO_14_) and brevetoxin B (C_50_H_70_O_14_). The most challenging is the structure of brevetoxin B (Fig. 20).

The highly complex molecular architecture is characterized by a novel array of ether oxygen atoms, regularly placed on a single carbon chain. This remarkable structure includes 11 rings, 23 stereogenic centers, and 3 carbon-carbon double bonds. The processing time necessary for running over all ~8.4 million stereoisomers corresponding to this structure was estimated to be about one month. The application of a genetic algorithm allowed us to correctly determine the *brevetoxin *stereochemistry and the 3D geometry model of the molecule (processing time of 2 h 50 m). This demonstrates the power of the approach we have described to facilitate the identification of relative stereochemistry in a complex molecule containing multiple stereocenters.

### System efficiency and future work

Different modes of system operating and manifold examples of its application to the molecular structure elucidation of new complex organic compounds (mostly natural products) have been described in our earlier articles [23-30,43,44]. These works contain detailed results of investigation of the system performance and working parameters. We have confirmed the high efficiency and applications of the StrucEluc system and the methodology of its application by the elucidation of the structures of more than 300 complex natural compounds. So far more than 100 large molecules with between 30 to 106 skeletal atoms have been elucidated using StrucEluc. The system is based on highly sophisticated, flexible and fast algorithms for structure generation, structure filtering and spectrum prediction. As a result, the total time for solving problems does not exceed one minute for > 80% of the problems solved. The system can interface to programs developed for the calculations of properties and physicochemical parameters of organic molecules from their structural formulae. This allows the prediction of many characteristics of the new compound elucidated using the system and can also generate systematic names according to IUPAC recommendations.

The StrucEluc system is a commercial product of Advanced Chemistry Development (ACD/Labs), and is presently widely used at many pharmaceutical companies and universities worldwide for the identification of newly isolated natural products, synthetic impurities, degradants and for the assignment of signals in 1-D and 2-D NMR spectra and the verification of structural hypotheses, etc.

## Conclusion

Computer-Aided Structure Elucidation is an area of chemoinformatics and analytical chemistry and has been developed over a period of forty years. This development path has forced the developers of CASE systems to overcome many obstacles hindering the development of a software application capable of drastically reducing the time and effort required to determine the structures of newly isolated organic compounds. Large complex molecules of up to 100 or more skeletal atoms with topological peculiarity can be quickly identified using, for example, the expert system Structure Elucidator based on spectral data. Logical analysis of 2D NMR data frequently allows for the detection of the presence of COSY and HMBC correlations of "nonstandard" length and provides a solution to the problem. Fuzzy structure generation provides a possibility to obtain the correct solution even in those cases when an unknown number of nonstandard correlations of unknown length are present in the spectra. The relative stereochemistry of big rigid molecules containing many stereocenters can be determined using the StrucEluc system and NOESY/ROESY 2D NMR data for this purpose.

The StrucEluc system is still being intensively developed in order to expand the general application, improved workflows and usability of the system and increased reliability of results. It is expected that expert systems similar to that described in this paper will receive increasing acceptance in the next decade and will ultimately be integrated directly to analytical instruments for the purpose of organic analysis. Work in this direction is in progress. In spite of the fact that many difficulties have already been overcome to deliver on the spectroscopist's dream of "fully automated structure elucidation" there is still work to do. Nevertheless, as the efficiency of expert systems is enhanced the solution of increasingly complex structural problems will be achievable.

## Abbreviations

CASE: Computer Aided Structure Elucidation; MCD: Molecular Connectivity Diagram; FF: Found Fragment(s); UF: User Fragment(s).

## Competing interests

The Structure Elucidator software program discussed in this publication is a commercial software product marketed by Advanced Chemistry Development (ACD/Labs). All authors are employees, ex-employees or collaborators of ACD/Labs.

## Authors' contributions

ME has been involved with the development of the Structure Elucidator software package for over a decade. AW was the product manager for Structure Elucidator during his employment with ACD/Labs and remains an active collaborator. KB is the project leader for Structure Elucidator. SM is the project leader for the structure generator component of the software. YS worked on the genetic algorithms associated with the stereochemical analysis. TC worked on database development, testing and validation of the software. All authors read and approved the final manuscript.
